# Influencing Factors of New Nurses’ Competency Following Participation in a Preceptorship Program: Cross-Sectional Study

**DOI:** 10.2196/75202

**Published:** 2025-11-13

**Authors:** Lusia Dian Wahyu Winarti, Krisna Yetti, Tuti Afriani, Enie Novieastari

**Affiliations:** 1 St Carolus Hospital, Jakarta, 10440, Indonesia; 2 Department of Fundamental of Nursing and Nursing Science, Faculty of Nursing, Universitas Indonesia, Kampus FIK UI, Jl. Prof. Dr. Bahder Djohan, Depok, Jawa Barat, 16424, Indonesia, 62 81332958572

**Keywords:** determinant, new nurse competency, preceptorship program, preceptor commitment, preceptor competency

## Abstract

**Background:**

Preceptorship programs have been implemented in several hospitals across Indonesia to support new nurses during their transition period in the workplace. Many factors influence new nurses successfully transitioning into this new role. However, few studies have examined the factors that affect new nurses’ competency.

**Objective:**

This study aimed to identify the factors influencing the competency of new nurses in a preceptorship program.

**Methods:**

This study used a quantitative approach with a cross-sectional design. Participants were 169 nurses who had been employed for less than 1 year in 2 hospitals. Participants were nurses undergoing an orientation period who were part of a preceptorship program. The study used instruments developed by the researchers and their team, which were tested for validity and reliability. The variables were self-efficacy, new nurses’ adaptation, preceptor commitment, preceptor competency, and mentoring method. Data were analyzed using descriptive statistics, the *χ*^2^ test, and multiple logistic regression.

**Results:**

The median age of the 169 participants was 24 years, with the ages ranging from 22 to 30 years. Most of the participants were female (n=136, 80.5%), held a bachelor’s degree (n=164, 97%), and had worked at Hospital X for 0 to 6 months (n=128, 75.7%). In terms of training experience, most participants had completed Basic Cardiac Life Support training (n=142, 84%). The independent variables that influenced new nurses’ competency were gender (*P*=.02), training (*P*=.05), mentoring method (*P*=.001), preceptor commitment (*P*=.03), and preceptor competency (*P*=.001). A multiple logistic regression test further indicated that the mentoring method (*P*=.001; *α*=.05; OR .198), preceptor commitment (*P*=.03; *α*=.05; OR .296), and preceptor competency (*P*=.001; *α*=.05; OR .202) were influential variables for new nurses’ competency.

**Conclusions:**

The mentoring method, preceptor commitment, and preceptor competency were identified as the factors that most strongly influence new nurses’ competency. These results can be used to develop more effective preceptor programs. An effective preceptorship program requires preceptors who demonstrate both professional competence and personal characteristics. Preceptors have to possess adequate knowledge and skills to support the development of new nurses’ competency.

## Introduction

Many factors influence the success of new nurses in undertaking a new role. A preceptorship program was designed to provide support for new nurses in their first year of delivering nursing care. The transition from student nurse to staff nurse is stressful for new nurses [1]. Work-related stress can occur in new nurses; therefore, efforts to minimize stress are a challenge for preceptors [2]. A study found that an environment with optimal learning and positive guidance provides the opportunity for students to acquire skills and receive regular feedback [3]. New nurses who cannot perform their competencies properly will reduce the quality of nursing services and patient safety in hospitals [4].

The transition from nursing student to staff nurse can be stressful or cause transition shock [5]. Factors that significantly affect transition shock are age, self-efficacy, work unit, desired unit, and the nurse’s work environment [6]. To reduce the transition shock of new nurses, it is necessary to provide a program to increase their self-efficacy [7]. Efforts should be made to ensure efficient human resource management and effective interventions by developing a global nursing competency improvement program based on nurse compassion competency and transcultural self-efficacy [8].

A previous study stated that preceptorship has an impact on learning and professional development in new nurses [9]. The orientation process requires a program that supports nurses through the transition phase, increasing self-confidence and competence [10]. New nurses have varied experiences, which extend to the quality of the orientation program and the role of the preceptor [11].

The preceptorship program is an opportunity for new nurses to develop and carry out assertive communication [12]. The competencies of a good preceptor include knowledge of individual learning processes, reflection skills, and giving effective feedback [13]. Well-trained preceptor nurses provide psychological stability to new nurses, increase job satisfaction, and promote organizational socialization [14]. The most important topics in nurse preceptor training were identified as critical thinking, prioritization, teaching techniques, conflict management, and teamwork [15]. In Indonesia, the preceptorship program has never been evaluated; besides that, there is no standardization of the preceptorship method, which is an obstacle to implementing the preceptorship program in hospitals. Another study found that preceptorship programs affected the work expectations, work environment, and turnover intention of new nurses [16].

## Methods

### Study Design

This study used a nonexperimental quantitative design with a cross-sectional approach. A self-report questionnaire was delivered by e-form at one point in time.

### Population

The population was new nurses at 2 hospitals with less than 1 year of service. Total sampling was used for this study. We selected these 2 hospitals because they are in the same city and have both implemented preceptorship programs. Total sampling was used to reach a larger number of respondents because the number of new nurses at one time in one hospital was not large enough. To reduce bias, we used enumerators to collect data.

Respondent inclusion criteria were new nurses who have worked for less than 1 year at hospitals. Respondents were undergoing an orientation period and preceptorship program. Respondents were only excluded if they were unwilling or absent when the data were collected.

### Data Collection

Respondents involved in this study were nurses from 2 hospitals. Hospital X had 107 nurses who participated and Hospital Y had 62 nurses.

### Measurement

The instrument is separated into 7 parts, namely respondent characteristics, self-efficacy [17], adaptation of new nurses, preceptorship methods [18], preceptor commitment [19], preceptor competence [20], and new nurses’ competency [21]. This instrument for new nurses’ competency was developed by the research team because we could not find a suitable instrument for new nurse competencies according to the standards in Indonesia. This instrument consists of 36 questions and has a validity value above the calculated *r* value, greater than the table *r* value (0.3). Meanwhile, Cronbach α was .989. We conducted a reliability test for new nurse self-efficacy (*r*=.934), new nurse adaptation (*r*=.878), preceptor competence (*r*=.996), preceptorship method (*r*=.948), and preceptor commitment (*r*=.807). The validity test is considered valid if the value of *r* count is greater than the table value of *r* (.361); therefore, all items were declared valid [22].

### Ethical Considerations

This research received ethical clearance from the Research Ethics Committee of the National Cardiovascular Center Harapan Kita Jakarta (letter number LB.02.01A/II/039/KEP039/2023).

## Results

### Demographic Characteristics

In this study, 169 new nurses participated, with a median age of 24 years. The youngest nurse was 22, while the oldest was 30. Most of the participants were female (n=136, 80.5%), had a bachelor’s degree (n=164, 97%), and had worked at a hospital for 0‐6 months (n=128, 75.7%). The nurses and midwives already had Basic Cardiac Life Support training (n=142, 84%; Table 1).

**Table 1.** Demographic characteristics of the new nurse respondents (N=169).

	Hospital X, n (%)	Hospital Y, n (%)	Total, n (%)
Median age (min-max)	24.0 (22–30)	25.0 (22–34)	24 (22-30)
Gender			
Male	23 (21.5)	10 (16.1)	33 (19.5)
Female	84 (78.5)	52 (83.9)	136 (80.5)
Education			
Bachelor degree	107 (100)	57 (91.9)	164 (97)
Diploma	0 (0)	5 (9.1)	5 (3)
Length of work (months)			
0‐6	106 (99.1)	22 (35.5)	128 (75.7)
6‐12	1 (0.9)	40 (64.5)	41 (24.3)
Training experiences			
BTCLS^a^	95 (88.8)	47 (75.8)	142 (84)
Patient safety	5 (4.7)	0 (0)	5 (3)
Other	7 (6.5)	15 (24.2)	22 (13)

a BTCLS: Basic Trauma Cardiac Life Support.

### Values of the Study Variables

Table 2 shows that the adaptation of new nurses and mentoring method had a lower median (both 2.9) than other variables (3.0), although the lowest minimum value was for preceptor commitment (1.5).

Researchers used the median value as a cutoff for determining whether each component was considered positive (more than the median score) or negative (less than the median score).

**Table 2.** Values of variables (N=169).

Variables	Items	Range	Median (min-max)
Self-efficacy	15	1-4	3.0 (2.4-4)
Adaptation of new nurses	24	1-4	2.9 (2.5-3.8)
Mentoring method	35	1-4	2.9 (2.2-3.9)
Preceptor commitment	8	1-4	3.0 (1.5-4)
Preceptor competence	95	1-4	3.0 (2.5-4)
New nurse competence	36	1-4	3.0 (2.6-4)

### Relationship Between the Preceptorship Program and New Nurse Competencies

As shown in Table 3, we found that the variables that were significantly related to the new nurse competencies were self-efficacy (*P*=.001), adaptation of new nurses (*P*=.001), mentoring methods (*P*=.001), preceptor commitment (*P*=.001), preceptor competence (*P*=.001), gender (*P*=.02), and training (*P*=.05).

**Table 3.** The relationship between the preceptorship program, demographic characteristics, and new nurse competencies (N=169).

	New nurse competencies			Odds ratio	95% CI	*P* value^a^
	Less, n (%)	Good, n (%)	Total, n (%)			
Self-efficacy (15 items)						
Negative (≤3.0)	68 (75.6)	22 (24.4)	90 (100)	10.475	5.1438‐21.356	<.001
Positive (>3.0)	18 (22.8)	61 (77.2)	61 (77.2)			
Adaptation of new nurses						
Negative (≤2.9)	61 (70.9)	25 (29.1)	86 (100)	5.36	2.924‐10.960	<.001
Positive (>2.9)	25 (30.1)	58 (69.9)	83 (100)			
Mentoring methods						
Negative (≤2.9)	71 (82.6)	23 (27.7)	94 (100)	12.348	5.917‐25.769	<.001
Positive (>2.9)	15 (17.4)	60 (72.3)	75 (100)			
Preceptor commitment						
Negative (≤3.0)	78 (90.7)	30 (36.1	108 (100)	17.225	7.330‐40.477	<.001
Positive (>3.0)	8 (9.3)	53 (63.9)	61 (100)			
Preceptor competence						
Negative (≤3.0)	72 (83.7)	19 (22.9)	91 (100)	17.23	8.036‐37.343	<.001
Positive (>3.0)	14 (16.3)	64 (77.1)	78 (100)			
Age						
≤25 y	5 (51.9)	51 (48.1)	106 (100)	1.113	.597‐2.077	.86
>25 y	31 (49.2)	32 (50.8)	63 (100)			
Gender						
Male	10 (30.3)	23 (69.7)	33 (100)	0.343	.152–.776	.02
Female	76 (55.9)	60 (44.1)	136 (100)			
Education						
Bachelor degree	84 (51.2)	80 (48.8)	164 (100)	1.575	.256‐9.674	.68
Diploma	2 (40)	3 (60)	5 (100)			
Length of work						
0‐6 mo	64 (50)	64 (50)	128 (100)	0.864	.427‐1.748	.82
6‐12 mo	22 (53.7)	19 (46.3)	41 (100)			
Training experiences						
BTCLS^b^	73 (51.4)	69 (48.6)	142 (100)	—^c^	—	.05
Patient safety	0 (0)	5 (100)	5 (100)			
Other	13 (59.1)	9 (40.9)	22 (100)			

a Significant if α<.05.

b BTCLS: Basic Trauma Cardiac Life Support.

c Not applicable.

### Factors Affecting the New Nurses’ Competency During the Preceptorship Program

As displayed in Table 4, we found in the final model that the independent variables (mentoring methods, preceptor commitment, and preceptor competence, gender, and training) simultaneously had a significant effect on the competence of new nurses (there must be at least 1 independent variable that influences the dependent variable, significant if α<.05).

**Table 4.** Factors affecting the new nurses’ competencies during the preceptorship program (N=169).

Variable	B	SE	Wald test	*P* value	Exp(B)	95% CI
Constant	1.583	0.679	5.441	.02	4.871	
Mentoring methods	–1.619	0.506	10.232	.001	0.198	0.073–0.534
Preceptor commitment	–1.216	0.56	4.711	.03	0.296	0.099–0.889
Preceptor competence	–1.599	0.5	10.239	.02	0.202	0.076–0.538
Gender	1.011	0.657	2.988	.08	2.75	0.873-8.657
Training	0.853	0.657	1.684	.19	2.346	0.647‐8.504

## Discussion

### Principal Findings

This study found that mentoring methods, preceptor commitment, preceptor competency, gender, and training were factors that influenced new nurses’ competency. A previous study stated that nurse mentors who have expertise and use a supportive approach can promote a healthy work environment [23]. Another study found 7 core competencies of nurse preceptors: teaching traits, clinical nursing profession, communication and collaboration, teaching pedagogy, reaction of contingency, critical thinking and reflection, and consultation on academic writing [24]. Previous research found that transition shock and perceptions of supervisors were significantly correlated with new nurses’ competency [25,26].

New nurses are expected to be able to adapt to the clinical environment, interact effectively, build strong partnerships with other professionals, and be able to make correct clinical decisions [27]. The mentor must be competent and have strong personal character traits and be able to think critically [28].

An important role of preceptors in the preceptorship program is to build new nurse competencies. A previous study stated that new nurses did not feel confident in performing many procedures independently without support from their supervisor (a manager, registered nurse, or mentor) [29].

In this study, the perception of new nurses regarding mentoring methods was still lacking (44.2% of new nurses gave a positive response to the mentoring method item). This was due to the lack of guidance in the orientation, classroom learning, mentoring, and evaluation phases. Another study found that updated guidance methods, including the application of evidence-based practice, can stimulate students to learn more about the cases they handle. This method can increase student knowledge [30]. In our study, the majority of preceptors had implemented the preceptorship method well, although there were still 18% who were not good [31].

The results of the statistical analysis showed that preceptor commitment had an effect on new nurse competencies. Another study found that there are low levels of role commitment among new mentors in Taiwan [32,33]. However, they act as good guides because this is an opportunity to teach, improve their teaching skills, share their knowledge, gain personal satisfaction, help new nurses and nursing students to integrate into the unit, and enhance their professional knowledge. A preceptor’s commitment to their role is associated with obtaining the benefits, appreciation, and support associated with this role. According to preceptors, to optimize the effectiveness of the nurse preceptorship program, it is necessary to understand that benefits, rewards, recognition, and support for preceptees must be an integral part of planning these programs [19].

### Implications and Limitations

An effective preceptorship program requires preceptors who have specific professional factors and personal characteristics. A clear evaluation process was planned from the start, incorporating feedback from preceptors and preceptees to make further improvements to the preceptorship program in the future. There is no standardization of preceptor competency; therefore, basic national competency standards for becoming a preceptor must be developed.

A limitation of this study is that the new nurse competency instrument was created by the researchers. This instrument is not yet standardized.

### Conclusions

Mentoring methods, preceptor commitment, preceptor competency, gender, and training were factors that influenced new nurses’ competency. Results from this research could be considered by hospitals’ nursing divisions when determining strategies to increase the competency of new nurses. Preceptorship programs are an important part of this strategy, where preceptor competence, preceptor commitment, and mentoring methods are a priority. New nurses must receive proper training to improve their competency before they enter the workforce.
